# Non-destructive identification of varieties of Hawaii-grown avocados using near-infrared spectroscopy: Feasibility studies using bench-top and handheld spectrometers

**DOI:** 10.1371/journal.pone.0303532

**Published:** 2024-06-06

**Authors:** Jemma M. Godbout, Nicolas C. Ladizinsky, Serenity Harris, Melissa L. Postler, Xiuxiu Sun, Tracie Matsumoto, Peishih Liang

**Affiliations:** 1 University of Hawaii at Hilo, Pacific Internship Program for Exploring Science, Hilo, Hawaii, United States of America; 2 USDA, Agricultural Research Service, U.S. Pacific Basin Agricultural Research Center, Hilo, Hawaii, United States of America; 3 University of Hawaii at Hilo, Department of Physics and Astronomy, Hilo, Hawaii, United States of America; Hainan University, CHINA

## Abstract

Avocados are an important economic crop of Hawaii, contributing to approximately 3% of all avocados grown in the United States. To export Hawaii-grown avocados, growers must follow strict United States Department of Agriculture Animal and Plant Health Inspection Service (USDA-APHIS) regulations. Currently, only the Sharwil variety can be exported relying on a systems approach, which allows fruit to be exported without quarantine treatment; treatments that can negatively impact the quality of avocados. However, for the systems approach to be applied, Hawaii avocado growers must positively identify the avocados variety as Sharwil with APHIS prior to export. Currently, variety identification relies on physical characteristics, which can be erroneous and subjective, and has been disputed by growers. Once the fruit is harvested, variety identification is difficult. While molecular markers can be used through DNA extraction from the skin, the process leaves the fruit unmarketable. This study evaluated the feasibility of using near-infrared spectroscopy to non-destructively discriminate between different Hawaii-grown avocado varieties, such as Sharwil, Beshore, and Yamagata, Nishikawa, and Greengold, and to positively identify Sharwil from the other varieties mentioned above. The classifiers built using a bench-top system achieved 95% total classification rates for both discriminating the varieties from one another and positively identifying Sharwil while the classifier built using a handheld spectrometer achieved 96% and 96.7% total classification rates for discriminating the varieties from one another and positively identifying Sharwil, respectively. Results from chemometric methods and chemical analysis suggested that water and lipid were key contributors to the performance of classifiers. The positive results demonstrate the feasibility of NIR spectroscopy for discriminating different avocado varieties as well as authenticating Sharwil. To develop robust and stable models for the growers, distributors, and regulators in Hawaii, more varieties and additional seasons should continue to be added.

## Introduction

The avocado tree (*Persea americana* Miller) belongs to the family Lauraceae and is an essential economic crop. Avocados from the Americas the West Indies were gradually introduced to Hawaii during the 1800s. With a harvested area of 820 acres, Hawaiiʻs avocado production ranks third among the states behind California and Florida, accounting for 3% of the total U.S. crop, amounting to a yield of 206,610 tons for the 2020–21 season [1]. While small in comparison to the total U.S. production, avocado remains an economically important crop for the state of Hawaii.

The avocado is botanically classified into three groups, Mexican, West Indian, and Guatemalan, with each displaying differences in fruit maturity and fat content [2]. Currently, there are over two hundred varieties of avocados grown in Hawaii, with Sharwil, a Mexican-Guatemalan cross originally from Australia, representing more than 57% of the commercial acreage in Hawaii [3, 4]. During the most recent season, roughly 600 tons of Sharwil avocados were produced and about 50 tons were exported to the continental United States [5]. In addition, three tons of other avocado varieties were reportedly shipped within the state, including Green Gold, Kahalu’u, Mālama, and San Miguel, which are well known for superior flavor, though grown in smaller percentages than Sharwil by commercial growers [6].

In 1992, the United States Department of Agriculture Animal and Plant Health Inspection Service (USDA-APHIS) rescinded the infestation-free quarantine procedure for Sharwil avocados after oriental fruit fly, *Bactrocera dorsalis* (Hendel) (Diptera: Tephritidae), larval infestation was found in fruits on trees in certified orchards in Kona, HI. Thereafter, all Hawaii-grown avocados were required to undergo quarantine treatments before export. The quarantine treatments such as methyl bromide and cold exposure negatively impacted fruit quality to a degree that Hawaii avocado growers would not export their crop, therefore losing out on U.S. mainland markets. In 2013, APHIS approved the resumption of export of only Sharwil avocados to the continental United States using a systems approach that confirmed the control of infestation by *B*. *dorsalis*. [4, 7]. The newly adopted regulations include registration with APHIS, grove sanitation, and orchard control such as APHIS approved lures and traps for *B*. *dorsalis*. Inspection of these traps with recorded data of *B*. *dorsalis* numbers and trap/lure location is required. Failure to comply with APHIS regulations could result in the termination or suspension of Sharwil shipments to the continental United States, resulting in over a million dollars of economic losses to the growers [8].

With the systems approach, Hawaii avocado growers must certify trees are Sharwil variety and export only Sharwil fruit. To confirm that an avocado is derived from a tree of a specific variety, the tree must be propagated asexually (vegetatively) from a known source, resulting in a clone of the intended cultivar [8]. Trees that are produced from the seeds of Sharwil avocados are the result of sexual recombination and may segregate and lose traits conferring resistance to fruit fly oviposition in the fruit. To identify Sharwil, growers rely on physical characteristics, such as color, size, and shape. The use of physical characteristics to positively identify a specific cultivar are not accurate, since many other varieties of avocados have cross traits displaying similar physical characteristics. In addition, molecular markers are used to clarify genetic relationships and fingerprint cultivars, but this method requires excised tissue samples from the skin which renders the fruit unmarketable. The misidentification of fruits may lead to Hawaii growers losing their valuable export markets as well as cause tensions between growers and authorities when the fruit is mislabeled. Developing a methodology that can identify Sharwil avocados accurately and non-destructively is critical to maintaining the economic viability of Hawaiian avocado growers.

Near-infrared (NIR) spectroscopy is a well-established method for the analysis of the chemical composition of organic materials. It is non-destructive, real-time, and can be relatively low cost. With a spectral range between 800-2500nm, NIR is ideal for the determination of chemical compounds containing O-H, C-H, and N-H bonds, due to the occurrence of overtone absorption bands of these functional groups within that spectral range [9, 10]. With shorter wavelengths (i.e. higher energy) as compared to mid-infrared, NIR takes advantage of deeper penetration in samples and is preferred when working with intact samples of commodities. NIR spectroscopy is routinely used for non-destructive determination of internal quality and/or maturity stages of apples, kiwifruits, grapes, persimmons, mangoes, etc. [11–15]. It has also been used to identify varieties and cultivars of Thai orange, Acai, peaches, and mango [16–20]. More specifically, it’s also demonstrated that NIR spectroscopy can be used to determine dry matter, water content, and fat content of avocados [8, 21–23].

The objective of this study is to evaluate the feasibility of NIR spectroscopy for non-destructive discrimination of avocado varieties and authentication of the Sharwil variety to satisfy quarantine requirements for Hawaii’s local and export markets.

## Materials and methods

### Avocado samples

931 avocados were acquired through the only packing house on the Island of Hawaii, Kane Plantation Avocados in Honaunau, from June 2022 through June 2023. Kane Plantation receives avocados from about 20 different commercial growers that produce avocados for export. Avocados were kept between 4°C and 6°C until one to eight days before spectral measurements. The selection of avocado varieties was based on seasonal availability. Sharwil (n = 537), collected in June, July, and September of 2022, as well as January and March of 2023, matures in winter and spring, has small seeds and greenish-yellow flesh with a rich, nutty flavor. Beshore (n = 52), collected between June and July of 2022, is a seedling of Sharwil and has similar high fat content. It is generally larger and more elongated, with dark green thick skin. Yamagata (n = 32), collected in June and July of 2022, is large and pear-shaped with a light green rough skin and a small seed. It is sometimes fibrous with a strong nutty flavor. Nishikawa (n = 97), collected in March 2023, is medium sized and pear-shaped with yellow flesh and high fat content. Greengold (n = 129), collected in February and March of 2023, is pyriform with a thick, green, pebbly skin, a small seed, yellow flesh, and high fat content. It was developed by the University of Hawaii and its taste is considered by some to be better than Sharwil. Two other non-commercial varieties were also collected in January and March 2023; one is colloquially known as Cannon ball (n = 43) for its shape and size and the other is an unnamed seedling of Sharwil (n = 41).

### NIR spectroscopy

A high-power tungsten halogen light source (Ocean Insight, Orlando, FL, USA) and a bench-top NIR spectrometer (NIRQuest+2.5, Ocean Insight, Orlando, FL, USA) with an InGaAs detector were used to measure adsorption of incident light on avocado samples at wavelengths from 900 to 2500 nm. Optical fibers were used to couple the light source, sample holder, and spectrometer. After ripening in room temperature for three days, 115 Sharwil, 52 Beshore, and 32 Yamagata from 2022 were measured at three random locations along the equatorial region using a desktop application (Ocean View 2.0, Ocean Insight, Orlando, FL, USA) with 50 ms integration time and a Boxcar width of 3 (i.e. 7-point averaging). To evaluate the feasibility of using less expensive and field-deployable tools, subsequent experiments were carried out using a handheld NIR spectrometer (F-750, Felix Instruments, Camas, WA, USA) measuring 2^nd^ derivative spectra between 729 nm and 975 nm. Spectra of additional 422 Sharwil, 129 Greengold, 97 Nishikawa, 43 Cannon ball, and 41 unnamed Sharwil seedling from 2023 were collected at three random locations along the equatorial region after ripening in room temperature for one, three, six, seven, and/or eight days.

### Chemical analysis

To validate the results from NIR spectroscopy, a subsample of 24 Sharwil, 18 Beshore, and 18 Yamagata from 2022 underwent destructive chemical analyses after spectral measurements. Dry matter (DM) of each avocado was determined individually in triplicates by drying 20 g samples at 102 °C to a constant mass. Dried avocado samples were then used to determine individual fat content using AOCS approved procedure Am 5–04 using an ANKOM extractor (XT10, ANKOM Technology, Macedon, NY, USA). Total antioxidant capacity of each avocado was determined in triplicate using a total antioxidant capacity assay kit (Sigma-Aldrich, St. Louis, MO, USA). Trolox solution was used for standard curves and absorbances at 570 nm were measured to determine concentrations using a 96-well microplate reader (SpectraMax M2, Molecular Devices, LLC, San Jose, CA, USA).

### Statistical analysis

All statistical analyses were performed using JMP Pro 16 (SAS, Cary, NC, USA). For the raw spectra measured by the bench-top spectrometer, Average spectrum per fruit was calculated between 1000 and 2400 nm, truncating the shortest and the longest 100 nm due to sensor limitations. Standard Normal Variate (SNV) preprocess was applied to the raw spectra to remove additive effects and multiplicative scatter. Tukey’s all pairwise comparison was performed to compare the means of dry matter, fat, and total antioxidant capacity between three varieties. For the 2^nd^ derivative spectra measured using the handheld spectrometer, average spectrum per fruit was calculated and no additional spectral preprocessing was applied. Canonical discriminant analysis was used to build calibration models using 60% of the samples (i.e. training set). Models were cross-validated and tuned using 20% of the samples and final classification rates were determined using the remaining 20% of the samples as test sets.

## Results and discussion

### Bench-top spectrometer: Sharwil, Beshore, and Yamagata

#### Spectra

**Fig 1:**
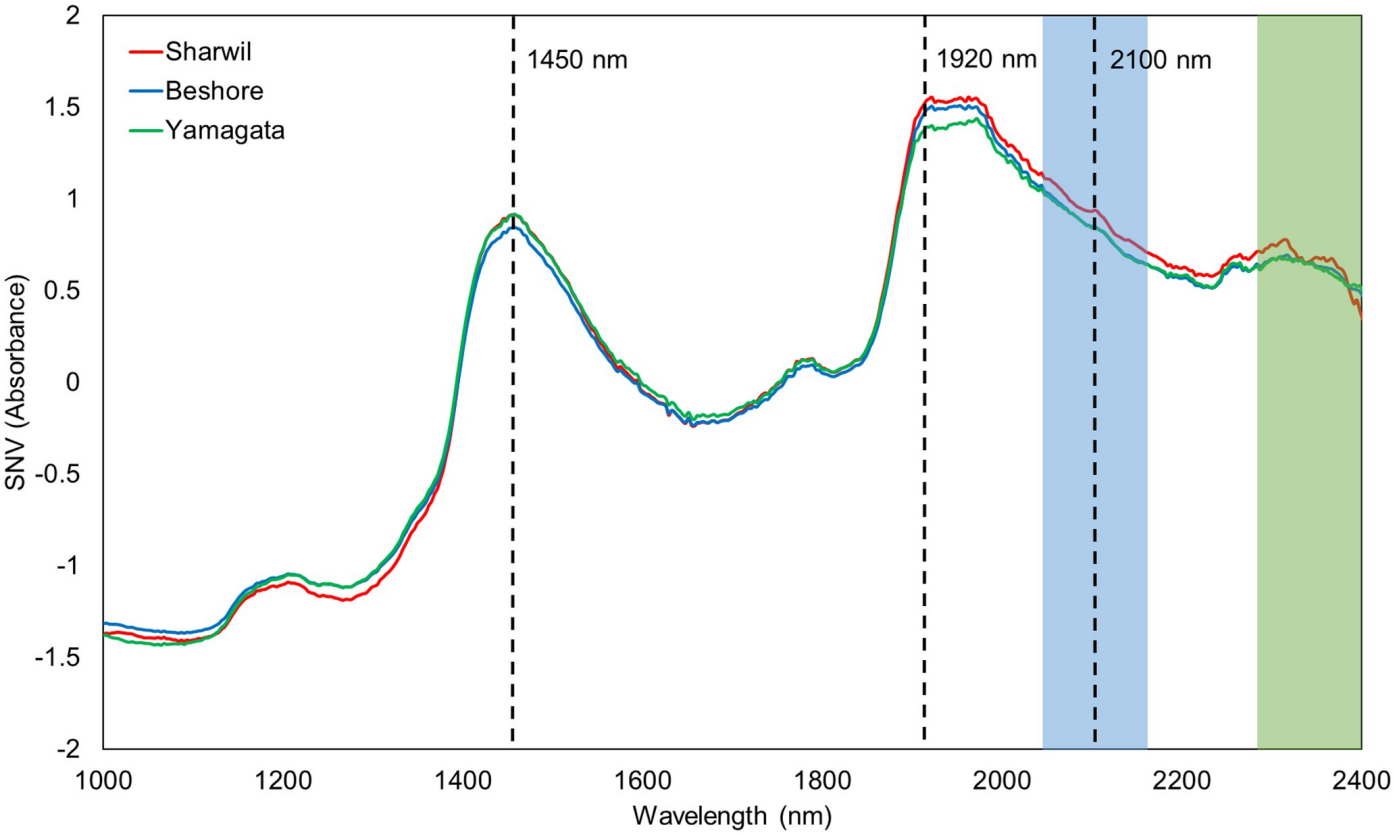
Average preprocessed spectra of 115 Sharwil, 52 Beshore, and 32 Yamagata from 2022.

Average SNV preprocessed spectra of Sharwil, Beshore, and Yamagata are shown in Fig 1. The three spectra have different signatures around 1450 nm and 1920 nm, which are known water absorption bands indicating internal quality differences in terms of dry matter. The same 1450 nm region may also include effects from protein (1485 nm) and cellulose (1460 nm and 1520 nm) [23, 24]. The three varieties also have visibly different spectra between 2050 nm and 2150 nm, which can be attributed to protein (2055 nm), starch (2100 nm), lipid (2142 nm), and cellulose (2079 nm and 2103 nm). The Sharwil variety can be seen separated from the other two at longer wavelengths, which could be attributable to its thinner skin compared to the other varieties. NIR at longer wavelengths have more limited penetration providing information relatively close to the surface, and there may be secondary correlations between skin properties and the long-wavelength region. Both Beshore and Yamagata have thicker and rougher skin than the Sharwil variety, which could contribute to the differences in spectra [23].

#### Canonical discriminant analysis

**Fig 2:**
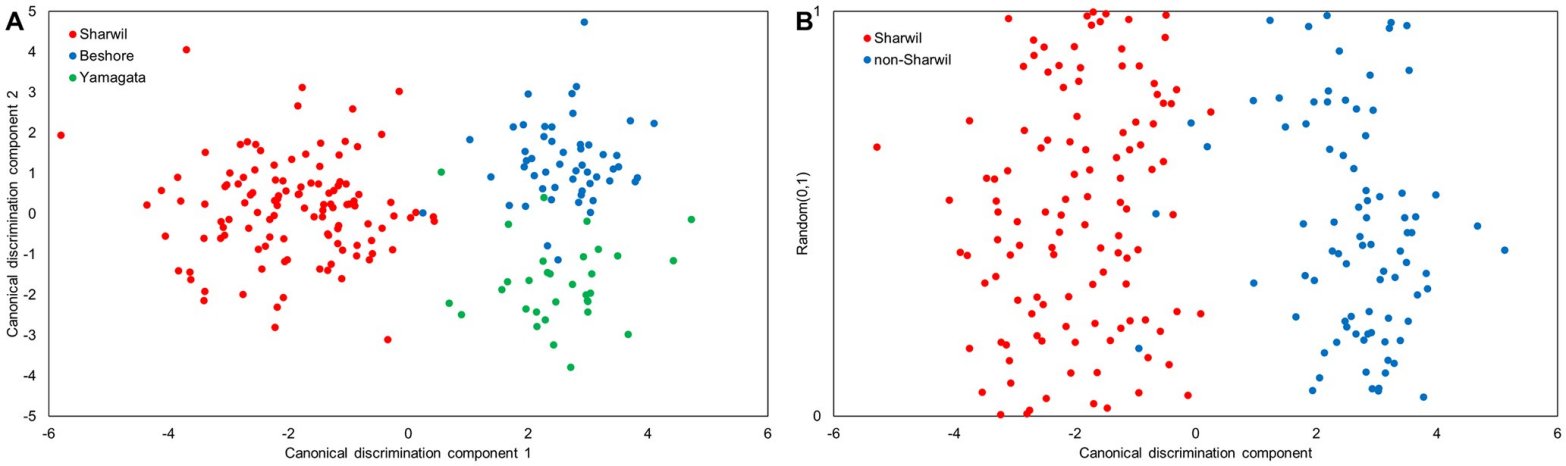
Canonical discriminant analysis plots for (A) discriminating between three varieties and (B) identifying Sharwil from the other two varieties, with a randomly generated y-axis to aid visualization.

Canonical discriminant analysis eliminates multicollinearity among the wavelengths and further reduce the dimensionality of the feature space by projecting the spectral data onto a two-dimension (for three classes) or a one-dimension (for two classes) plot that provides maximal separation between groups (Fig 2). The plots clearly visualize the separation between groups and with Fig 2A, it shows that canonical discrimination component 1 separates Sharwil from the other two varieties and canonical discrimination component 2 further discriminates between Beshore and Yamagata.

Canonical discriminant analysis results show 95.8%, 92.5%, and 95% total classification rates (i.e. accuracy) for the training, validation, and test sets, respectively, to discriminate between the three varieties. The classifier for identifying Sharwil from the other two varieties achieves 99.2%, 100%, and 95% total classification rates for the training, validation, and test sets, respectively. The classification matrices of the test sets with the numbers of false positives and false negatives are shown in Table 1. The precision of our test set model in identifying Sharwil is 95.7% when against the other two varieties, calculated using the following formula:

$$Precision=\frac{True\;Positives}{True\;Positives+False\;Positives}$$

**Table 1:** Classification matrices of the test sets for discriminating three varieties and identifying Sharwil from the other two varieties.

Actual	Predicted (% classification)			Actual	Predicted (% classification)	
	Sharwil	Beshore	Yamagata		Sharwil	Others
Sharwil	23 (100%)	0	0	Sharwil	22 (95.7%)	1 (4.3%)
Beshore	0	10 (90.9%)	1 (9.1%)	Others	1 (5.9%)	16 (94.1%)
Yamagata	0	1 (16.7%)	5 (83.3%)			

The accuracy and precision reported here are on par with the performances reported by other studies authenticating varieties of mangoes and peaches [19, 20, 25].

**Fig 3:**
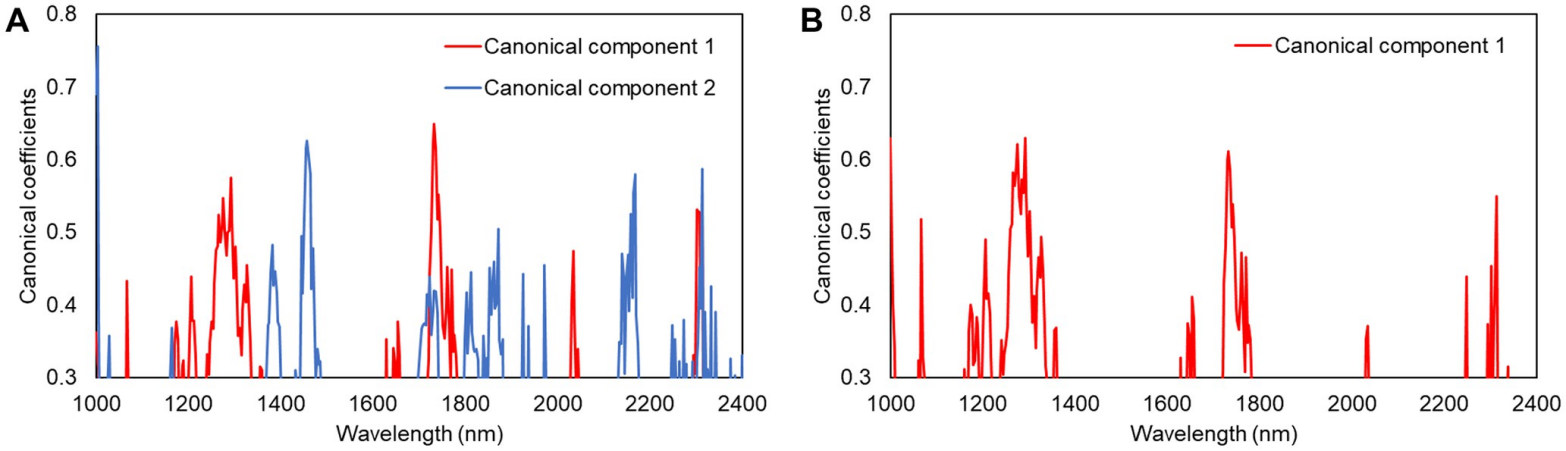
Canonical coefficient plots show the contribution of each variable in the classifier (A) discriminating three varieties and (B) identifying Sharwil from the other two varieties.

To assess the importance of each variable, standardized canonical coefficients of the two canonical components to discriminate three varieties as well as the one canonical component to identify Sharwil from the other two varieties were evaluated. Variables with standardized coefficients greater than 0.3 are shown in Fig 3. The coefficients for the first canonical component of the three-variety classifier and the coefficients for the one canonical component of the two-variety classifier are nearly identical because both components were computed to separate Sharwil from the other two varieties.

Contrary to the observations made based on the average spectra (Fig 1), the variables that are influential in discriminating Sharwil from the other varieties do not align with the water bands at 1450 nm and 1920 nm, instead they mostly align with known lipid and protein bands. The water bands are more influential in canonical component 2 to discriminate Beshore and Yamagata.

#### Dry matter, fat content and total antioxidant capacity

The DM content, fat content, and total antioxidant capacity of a subset of 24 Sharwil, 18 Beshore, and 18 Yamagata were measured through July 2022 and the results are shown in Table 2. The DM contents of Beshore (39.9 ± 0.2%) and Sharwil (40.2 ± 0.4%) are significantly higher than that of Yamagata (31.2 ± 0.4%). DM content is associated with fatty acid content, which differs among avocado varieties as well as harvesting stages. For example, the DM content of Hass avocado is between 30 and 37%, depending on the harvest time, and the DM content of Reed avocado was reported to increase by 33% from the early to the late harvesting stage [26–28]. DM content is also affected by respiration rate and increased ripening, and high levels of fat are concentrated in the pulp at the expense of DM during ripening [29]. Storages at cold room temperatures (5 °C and 10 °C) are reported to considerably slow down the metabolic activities of avocado fruits, thus slowing down the decrease in dry matter in the same storage period [26]. All 60 avocados analyzed were harvested mature, stored between 4°C and 6°C, and left to ripen at room temperature for 3 days before measurements, to minimize the variations caused by different conditions.

**Table 2:** The mean ± standard errors of dry matter, fat content and total antioxidant capacity of 60 avocados. Different lower-case letters denote significant differences in means between the groups.

Variety	Dry Matter (%)	Fat Content (%)	Total Antioxidant Capacity (nmol/μL)
Sharwil	40.2 ± 0.4^a^	33.1 ± 0.7^a^	26.0 ± 0.9^a^
Beshore	39.9 ± 0.2^a^	36.2 ± 0.6^a^	22.1 ± 0.6^a^
Yamagata	31.2 ± 0.4^b^	22.1 ± 0.8^b^	23.4 ± 0.6^a^

The fat contents of Beshore (36.2 ± 0.6%) and Sharwil (33.1 ± 0.7%) were significantly higher than Yamagata (22.1 ± 0.8%) (Table 2). Like DM contents, fat contents also vary among varieties and harvesting stages. The fat contents of Fuerte, Hass, Pinkerton, and Reed avocado are between 16–25% depending on cultivars and harvesting stages, increasing by around 31% from the early to the late harvest stage [28, 30]. The similarity between Sharwil and Beshore is expected as Beshore is a seedling of Sharwil and is similar in biochemical compositions.

Avocado fruit is an extremely rich source of bioactive phytochemicals, including C7 sugars, sterols, carotenoids, vitamin E, and other antioxidant compounds [31]. The total antioxidant capacity for the three varieties ranged from 22.1 to 26.0 nmol/μL, with Sharwil showing slightly higher total antioxidant capacity than Beshore and Yamagata though not statistically significant (Table 2).

Although the exact correlation between different compounds and their contributions to the classifiers is difficult to establish due to the overlapping nature of NIR spectra as well as the small sample size of the subsample that were analyzed destructively, the chemical analysis partially validated our spectral results that lipid and water are two influential factors in discriminating avocado varieties.

### Handheld spectrometer: Sharwil, Greengold, Nishikawa, and others

#### Spectra

**Fig 4:**
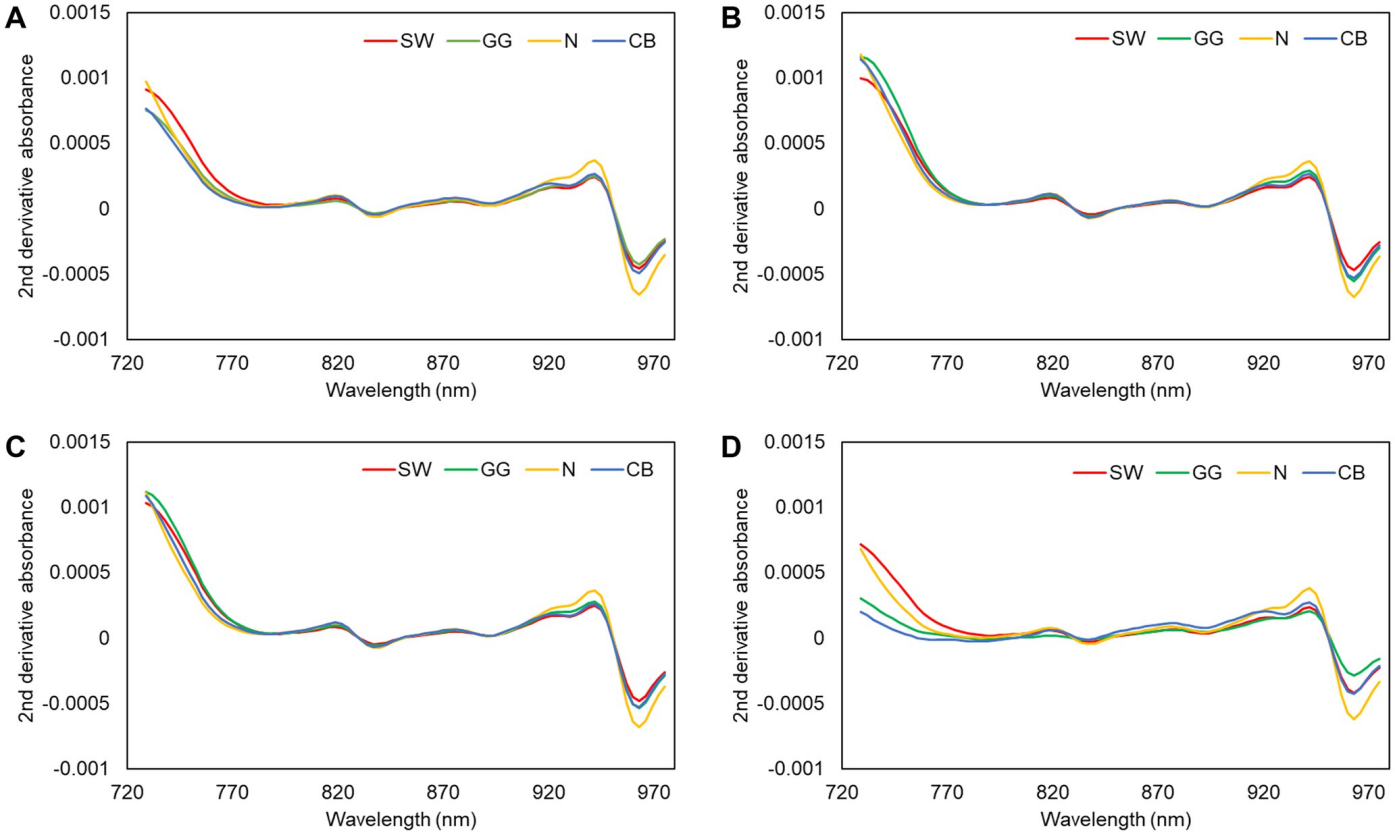
The average spectra of 422 Sharwil (SW), 129 Greengold (GG), 97 Nishikawa (N), 43 Cannon ball (CB) from 2023 (A) across all ripening stages, and at (B) one, (C) three, and (D) more than six days.

The avocados, except for 41 unnamed Sharwil seedling and 27 Sharwil, were kept in room temperature and spectra were collected after one, three, six, seven, and/or eight days. Total of 1991 spectra were collected across three commercial varieties (Sharwil, Greengold, and Nishikawa) and two non-commercial varieties (Cannon ball and unnamed seedling Sharwil) and across all ripening stages. The average spectra of the three commercial varieties and Cannon ball across all ripening stages, and at one, three, and more than six days are shown in Fig 4.

It can be seen from Fig 4 that Nishikawa has more distinct spectra from the other varieties, and the difference between varieties also become more apparent after six or more days in room temperature.

#### Canonical discriminant analysis

Canonical discriminant analysis results show 97.7%, 97.5%, and 96% total classification rates for the training, validation, and test sets, respectively, to discriminate between the five varieties regardless of the ripening stages. The classifier for identifying Sharwil from the other four varieties achieves 96.4%, 97.2%, and 96.7% total classification rates for the training, validation, and test sets, respectively. The precision of our model in identifying Sharwil is 98.2% against the other four varieties. Table 3 shows classification matrices of the test sets with the numbers of false positives and false negatives.

**Table 3:** Classification matrices of the test sets for discriminating five varieties and identifying Sharwil from the other four varieties.

Actual	Predicted (% classification)					Actual	Predicted (% classification)	
	Sharwil	Greengold	Nishikawa	Canon ball	Sharwil seedling		Sharwil	Others
Sharwil	218 (95.6%)	9 (3.9%)	0	0	1 (0.4%)	Sharwil	222 (97.8%)	5 (2.2%)
Greengold	3 (5.8%)	49 (94.2%)	0	0	0	Others	8 (4.7%)	163 (95.3%)
Nishikawa	0	1 (1.3%)	76 (98.7%)	0	0			
Canon ball	0	1 (3%)	0	32 (97%)	0			
Sharwil seedling	1 (12.5%)	0	0	0	7 (87.5%)			

**Fig 5:**
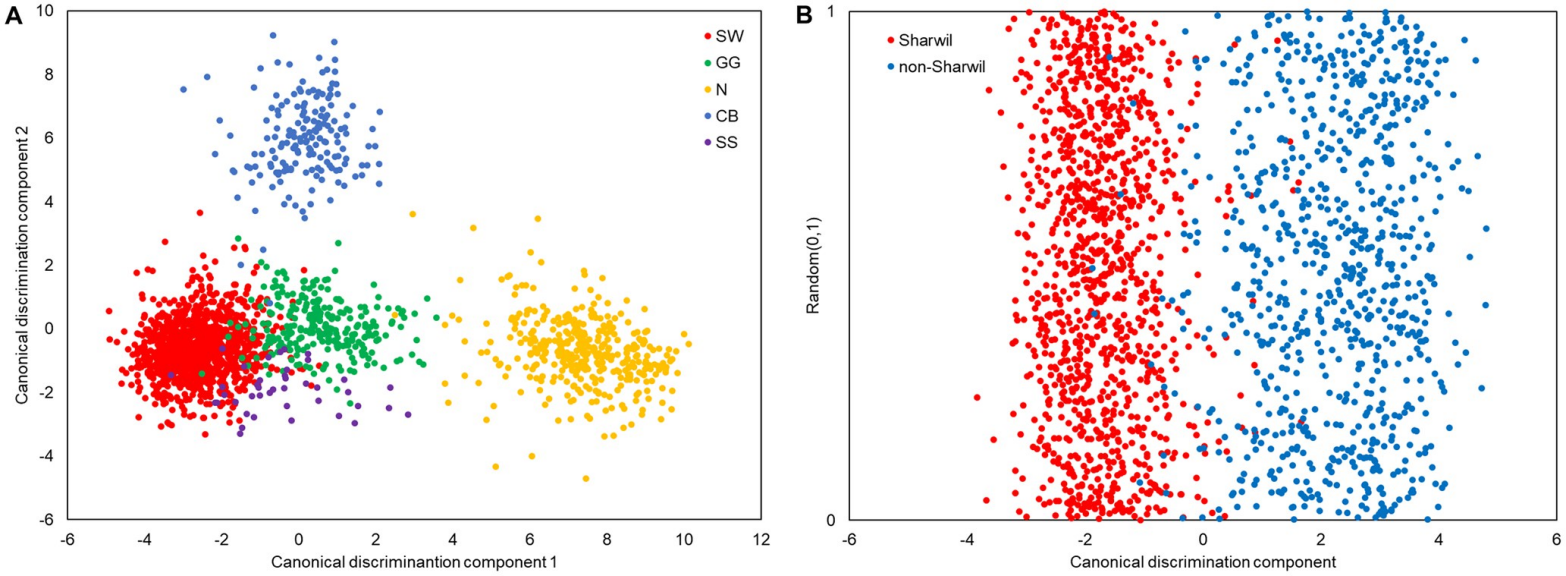
Canonical discriminant analysis plots for (A) the first two dimensions in discriminating between five varieties and (B) identifying Sharwil from the other four varieties.

Canonical discriminant analysis reduces the dimensionality of the feature space by projecting the spectral data onto four dimensions (for five classes) or one dimension (for two classes) that provide maximal separation between groups. Fig 5A shows the first two dimensions in separating the five varieties. Canonical discrimination component 1 separates Nishikawa from the other four varieties and canonical discrimination component 2 further discriminates Cannon ball from the remaining three. Fig 5B shows the separation between Sharwil and the other varieties with a randomly generated y-axis to aid visualization.

With more varieties at different ripening stages, the classifiers perform very well even with a limited spectral range (729 nm– 975 nm) using a handheld spectrometer. This indicates that the differences in biochemical compositions between varieties of avocados are greater than the biochemical changes overtime as the fruits ripen and can be measured non-destructively in real-time through NIR spectroscopy.

## Conclusions

Of over 200 varieties of Hawaii-grown avocados, currently only the Sharwil variety can be exported to the continental United States with a systems approach without quarantine treatments. Hawaiian growers must follow strict USDA-APHIS regulations and confirm the avocados are of Sharwil identification with APHIS before export. This study evaluates the feasibility of using two NIR systems to discriminate Hawaii-grown avocado varieties and to positively identify Sharwil avocados from the other varieties, to improve or aid the current methods for variety identification. Best result achieved using a bench-top system shows a 95% total classification rate in positively identifying Sharwil from two other varieties. Through observing average spectra plots and evaluating predictor importance, wavebands associated with fat, protein, cellulose, and starch are the compounds contributed the most to the classifiers. Results from chemical analysis done on a subset of avocados also partially validated the successful classifications. Furthermore, a 96.7% total classification rate was achieved in positively identifying Sharwil from four other varieties using a handheld spectrometer with no detectable damage to the marketability of the fruits. The feasibility of using NIR to discriminate avocado varieties and to authenticate Sharwil avocado is demonstrated. Future efforts to add more varieties from different seasons can aid the development of a spectral library of Hawaii avocados that will provide a more efficient, reliable, non-destructive, and cost-effective method for growers, distributors, and regulators in Hawaii.
